# Upregulated galectin-1 in *Angiostrongylus cantonensis* L5 reduces body fat and increases oxidative stress tolerance

**DOI:** 10.1186/s13071-022-05171-4

**Published:** 2022-02-05

**Authors:** Wei-Wei Sun, Xiu-Mei Yan, Ai-Jun Qiao, Yuan-Jiao Zhang, Ling Yang, Hui-Cong Huang, Hong-Fei Shi, Bao-Long Yan

**Affiliations:** 1 Department of Parasitology, School of Basic Medical Sciences, Wenzhou Medical University, Wenzhou, 325035 Zhejiang People’s Republic of China; 2 Department of Pediatric Gastroenterology, The Second Affiliated Hospital and Yuying Children’s Hospital of Wenzhou Medical University, Wenzhou, 325000 Zhejiang People’s Republic of China; 3 Department of Biochemistry, School of Basic Medical Sciences, Wenzhou Medical University, Wenzhou, 325035 Zhejiang People’s Republic of China; 4 Department of Biomedical Engineering, School of Medicine and School of Engineering, University of Alabama at Birmingham, Birmingham, AL 35294 USA; 5 Henan Provincial Engineering Laboratory of Insects Bio-Reactor, China-UK-NYNU-RRes Joint Laboratory of Insect Biology, Nanyang Normal University, Nanyang, 473061 People’s Republic of China

**Keywords:** *Angiostrongylus cantonensis*, Galectin-1, *Caenorhabditis elegans*, Oxidative stress, Fat

## Abstract

**Background:**

*Angiostrongylus cantonensis* L5, parasitizing human cerebrospinal fluid, causes eosinophilic meningitis, which is attributed to tissue inflammatory responses caused primarily by the high percentage of eosinophils. Eosinophils are also involved in killing helminths, using the peroxidative oxidation and hydrogen peroxide (H_2_O_2_) generated by dismutation of superoxide produced during respiratory burst. In contrast, helminthic worms have evolved to attenuate eosinophil-mediated tissue inflammatory responses for their survival. In previous study, we demonstrated the extracellular function of *Acan*-Gal-1 in inducing the apoptosis of macrophages. Here, the intracellular functions of *Acan*-Gal-1 were investigated, aiming to further reveal the mechanism involved in *A. cantonensis* L5 worms surviving inflammatory responses in the human central nervous system.

**Methods:**

In this study, a model organism, *Caenorhabditis elegans*, was used as a surrogate to investigate the intracellular functions of *Acan*-Gal-1 in protecting the worm from its host’s immune attacks. First, structural characterization of *Acan*-Gal-1 was analyzed using bioinformatics; second, qRT-PCR was used to monitor the stage specificity of *Acan-gal-1* expression in *A. cantonensis*. Microinjections were performed to detect the tissue specificity of *lec-1* expression, the homolog of *Acan-gal-1* in *C. elegans*. Third, microinjection was performed to develop *Acan-gal-1::rfp* transgenic worms. Then, oxidative stress assay and Oil Red O fat staining were used to determine the functions of *Acan*-Gal-1 in *C. elegans*.

**Results:**

The results of detecting the stage specificity of *Acan-gal-1* expression showed that *Acan*-Gal-1 was upregulated in both L5 and adult worms. Detection of the tissue specificity showed that the homolog of *Acan-gal-1* in *C. elegans*, *lec-1* was expressed ubiquitously and mainly localized in cuticle. Investigating the intracellular functions of *Acan*-Gal-1 in the surrogate *C. elegans* showed that N2 worms expressing *pCe-lec-1::Acan-gal-1::rfp*, with lipid deposition reduced, were significantly resistant to oxidative stress; *lec-1* mutant worms, where lipid deposition increased, showed susceptible to oxidative stress, and this phenotype could be rescued by expressing *pCe-lec-1::Acan-gal-1::rfp*. Expressing *pCe-lec-1::Acan-gal-1::rfp* or *lec-1* RNAi in *fat-6;fat-7* double-mutant worms, where fat stores were reduced, had no significant effect on the oxidative stress tolerance.

**Conclusion:**

In *C. elegans* worms, upregulated *Acan-*Gal-1 plays a defensive role against damage due to oxidative stress for worm survival by reducing fat deposition. This might indicate the mechanism by which *A. cantonensis* L5 worms, with upregulated *Acan-*Gal-1, survive the immune attack of eosinophils in the human central nervous system.

**Graphical Abstract:**

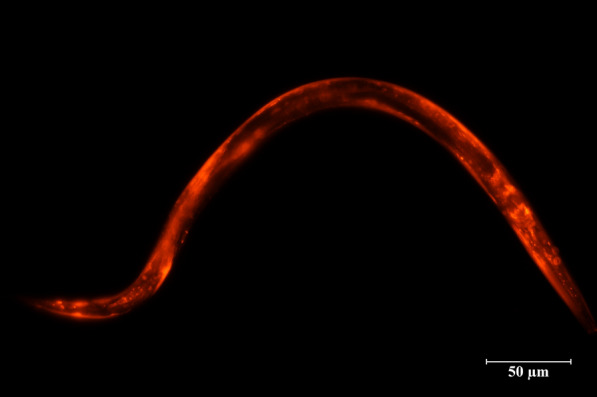

**Supplementary Information:**

The online version contains supplementary material available at 10.1186/s13071-022-05171-4.

## Background

*Angiostrongylus cantonensis* is considered the primary causative pathogen of human eosinophilic meningitis and meningoencephalitis in China, Japan, Southeast Asia and the Pacific Islands [1–3]. The final host of this parasite is rat; it lives in the rat pulmonary artery where it develops to sexual maturity [4, 5]. Human, as an atypical host, mainly acquires this parasite by consuming raw terrestrial freshwater snails, such as the golden apple/channeled apple snail *Pomacea canaliculata*, in which the infective third-stage larvae (iL3) reside. After passage to the human small intestine, iL3 will infect the central nervous system through the bloodstream and develop into fifth-stage larvae (L5), causing eosinophilic meningitis [6–10].

High percentage of eosinophils, recruited from the circulation into the central nervous system [7], as the primary pathological change in eosinophilic meningitis, is thought to contribute to tissue inflammatory responses and host defense in helminthic infections [11]. High level of the enzyme complex is expressed and released in the granule matrix of eosinophils, such as eosinophil peroxidase [12, 13], which is associated with helminthic killing, using the peroxidative oxidation and hydrogen peroxide (H_2_O_2_) generated by dismutation of superoxide produced during respiratory burst [14–16]. In contrast, helminthic worms, parasitizing with a high level of eosinophils, have evolved to attenuate eosinophil-mediated tissue inflammatory responses for their survival [11]. Therefore, *A. cantonensis* L5, residing in human cerebrospinal fluid (CSF) with a high percentage of eosinophils, may act to resist the oxidative stress damage from eosinophils.

In our previous study, proteomic analysis of *A. cantonensis* L5 and *A. cantonensis* iL3, using two-dimensional difference gel electrophoresis (2D-DIGE), showed that the expression level of *A. cantonensis* RPS-30 (*Acan*-RPS-30) was lower in L5 than that in iL3 and that of *A. cantonensis* Galectin-1 (*Acan*-Gal-1) was higher [8]. Recently, we have demonstrated that downregulated *Acan-*RPS-30 in *A. cantonensis* L5 plays a defensive role in worm survival against damage due to oxidative stress by inhibiting apoptosis by regulating *ced-3* downregulation [17].

*Acan*-Gal-1, as the homologous protein of human Galectin-9 and *Caenorhabditis elegans* LEC-1, belongs to the family of galectins that are glycan-binding proteins distributed among animals and fungi and is defined by its conserved carbohydrate recognition domains (CRDs) and affinity for β-galactoside structures [18–20]. Galectins are secreted by cells via an unconventional mechanism [21, 22] and function in various biological phenomena, such as development, immunity and tumorigenesis via recognition of cell surface or extracellular glycoconjugates [22]. Parasite galectins have a sequence and structure similar to those of mammalian homologs and are presumed to participate in host-parasite interactions [23, 24]. Our previous studies showed that upregulated *Acan*-Gal-1 in *A. cantonensis* L5 induced the apoptosis of macrophages by binding to Annexin A2 and activating the JNK apoptotic signaling pathway [24]. However, galectins are also found intracellularly and are involved in RNA splicing, cell growth, apoptosis and other functions [25, 26]. In the nematode *C. elegans*, 11 galectins have been determined, and the endogenous ligands and some of the functions of LEC-6, LEC-8 and LEC-10 have been identified. LEC-6 has a role in growth regulation by affecting the localization and function of its ligands such as F57F4.4 [27, 28]; LEC-8 acts against bacterial infection by binding to glycolipids [29], and LEC-10 functions against oxidative stress by binding to glycoproteins [27, 30]. LEC-1, the homolog of *Acan*-Gal-1, is a tandem repeat-type galectin, and its two CRDs have different sugar-binding properties [31]. As LEC-1 is mainly localized in the cuticle and pharynx of *C. elegans* [32], it is thought to have some functions as a component of the durable outer barrier via recognizing and cross-linking glycoconjugates by its two CRDs with different sugar-binding properties and to play a defensive role against damage due to oxidative stress in *C. elegans* [33]. Therefore, *Acan*-Gal-1, upregulated in *A. cantonensis* L5, may have the function of resisting human immune attacks by not only inducing the apoptosis of its host’s macrophages extracellularly [24], but also increasing oxidative stress tolerance of the worm itself intracellularly.

In this study, we further explored the intracellular functions of *Acan*-Gal-1 upregulated in *A. cantonensis* L5, using the model organism *C. elegans* as a surrogate, in relation to the lack of effective genetic manipulation in parasitic nematodes, to investigate its role in protecting the worm from its host’s immune attacks.

## Methods

### Propagation of *A. cantonensis* and *C. elegans*

*Angiostrongylus cantonensis* ZJ strain was maintained and propagated in Wenzhou Medical University, China, by cycling through *Pomacea canaliculata* and Sprague-Dawley (SD) rats as described previously [8]. Intermediate hosts *P. canaliculata* were infected with *A. cantonensis* L1 by feeding on rat feces. L3 were collected at 20 days p.i. Infected snails were shelled and crushed. The intestines and other organs were removed, and the remaining tissue was homogenized. The homogenates were filtered through a 40-mesh sieve, deposited for 5 min at 4 °C and precipitated 2–3 times at room temperature. The sediments were removed, and L3 number and viability were determined by direct observation under a light microscope. Three-week-old Sprague-Dawley (SD) rats [weight 100–120 g, grade clean, Certificate SYXK (zhe2015-0009)], supplied by the Laboratory Animal Center of Wenzhou Medical University, were orally infected with 50 L3/rat. The rats were housed in polypropylene cages with free access to food and water and then killed by anesthesia at 25 days and 45 days p.i., respectively. The L3 worms were collected from the intermediate host *P. canaliculata* and L5 harvested from the brains of mice [C57BL/6J (B6), Certificate SYXK (zhe2015-0009)] (non-permissive host same as humans), which were orally infected with 30 L3/mouse. Adult worms were collected from the blood vessels of the hearts and lungs. Individuals of different sexes were separated using morphological criteria: females are usually longer and thinner than males, and males exhibit typical copulatory bursa. L3, L5 and adults were washed three times with 0.01 mol/l PBS buffer and stored at − 80 °C. These rats were not used for any other part of the study.

*Caenorhabditis elegans* strains N2, *lec-1* (*tm1345*), *ced-3* (*ok2734*) and *fat-6;fat-7* (BX156) were maintained on Nematode Growth Media (NGM) agar plates at 20 °C as described previously [34]. Worms were fed with *Escherichia coli* strain OP50 unless otherwise stated. The mutant strains *lec-1* (*tm1345*), *ced-3* (*ok2734*) and *fat-6;fat-7* (BX156) were obtained from the Caenorhabditis Genetic Center (CGC) (University of Minnesota, USA).

### Isolation, purification, treatment and storage of nucleic acids

Total RNA was extracted from worms at different developmental stages employing Trizol reagent (Invitrogen, USA), followed by treatment with 2 U of deoxyribonuclease I (DNase I; Takara Biotechnology Co., Ltd, Japan). First-strand cDNA was obtained using the Murine leukemia virus reverse transcriptase (M-MLV RTase cDNA Synthesis Kit; Takara Biotechnology Co., Ltd, Japan). RNA samples were stored at − 80 °C.

### Bioinformatic analysis

The homolog of *Acan*-Gal-1in *C. elegans*, LEC-1 (GenBank: NP_001370038.1), was selected to align with *Acan*-Gal-1 using Clustal Omega (http://www.clustal.org/omega/). Homology models were built by SWISS-MODEL (https://swissmodel.expasy.org/), using *Toxascaris leonina Tl*-Gal-9 (PDB code 5glv.1; Protein Data Bank, https://www.rcsb.org/) [35] as template. Three-dimensional structural analysis was performed using the PyMOL molecular viewer program. All calculations were carried out under default conditions.

### Quantitative real-time PCR (qRT-PCR) analysis

qRT-PCR was performed to determine the abundance of *Acan-gal-1* transcripts in different developmental stages (L3, L5 female, L5 male, adult female, adult male) of *A. cantonensis*.

Gene expression levels were determined by qRT-PCR using SYBR®Green PCR Master Mix and a 7500 Real-Time PCR System (Applied Biosystems, USA). Relative gene expression was compared with 18S ribosomal RNA gene (GenBank: AY295804) as an internal loading control. The target genes and primers used are listed in Additional file 1: Table S1. Statistical analysis was conducted using a one-way ANOVA; *P* < 0.05 was set as the criterion for significance.

### Transgenic worms

An about 2000-bp sequence upstream of *Acan-gal-1* 5′ untranslated region (5′-UTR) was used as putative promoter. To analyze promoter activity of *Acan-gal-1*, the promoter regions of *Acan-gal-1* and *Ce-lec-1* were amplified and cloned into plasmid pPD95.77 to construct *pAcan-gal-1::gfp* and *pCe-lec-1::rfp*, respectively.

To perform cross-species expression of *Acan*-Gal-1 in *C. elegans* strains, cDNA sequence was amplified and cloned into pPD95.77 using the promoter of *Ce-lec-1* to construct plasmid *pCe-lec-1::Acan-gal-1::rfp*. All primers used are listed in Additional file 1: Table S1.

All plasmid constructs were confirmed by sequencing.

Recombinant plasmids were each microinjected into the gonads of young adult *C. elegans* hermaphrodites as described [6] together with plasmid pRF4 containing a dominant mutant allele of *rol-6* gene, each at a final concentration of 50 μg/ml in the same mixture, using pPD95.77 (*pCe-lec-1::rfp*) and pRF4 plasmid mixture as a control. Plasmid pRF4 was included in all transformations as a behavioral marker, and transgenic worms showing the roller phenotype were selected. F2 and subsequent generations were analyzed and selected to examine the expression patterns of GFP or RFP using a fluorescent microscope (Olympus IX71). A minimum of three independent lines expressing each transgene was evaluated*.*

### Oxidative stress assay

The oxidative stress assay was performed as described previously [30]. Briefly, adult hermaphrodites (30 worms/group) were transferred to a 96-well plate containing 3 mM H_2_O_2_ in M9 buffer (M9 buffer: 3 g/l KH_2_PO_4_, 6 g/l Na_2_HPO_4_, 5 g/l NaCl and 0.12 g/l MgSO_4_). After incubation at 20 °C for the specified durations, the number of dead worms was determined. Worms were scored as dead when they no longer responded with movement to light prodding of the head. Three (H_2_O_2_) independent experiments were performed. Statistical analysis was performed with Microsoft Excel 2010 software using an unpaired two-tailed *t*-test.

### RNAi feeding experiments

RNAi experiments were performed with feeding worms, *E. coli* strain HT115 (DE3), transformed with either the empty vector L4440 as the control or *lec-1*gene-targeting constructs from the *C. elegans* Ahringer RNAi Collection [36]. To generate *lec-1*-specific RNAi vectors, *lec-1* partial cDNA was cloned into the L4440 vector. Plasmids were transformed into *E. coli* strain HT115. Primers used for PCR analysis are listed in Additional file 1: Table S1. RNAi plates and media were prepared according to [17]. Briefly, overnight bacterial culture in LB supplemented with ampicillin (100 mg/ml) at 37 °C was seeded onto NGM plates containing IPTG (1 mM) and ampicillin (100 mg/ml) and incubated overnight at room temperature to induce double-stranded RNA production. Gravid adult *ced-3* mutant worms and *fat-6;fat-7* double-mutant worms were allowed to lay eggs overnight on the RNAi plates, and adult worms were picked off. Embryos were placed on RNAi plates and incubated at 20 °C until adulthood to score phenotypes.

### Lipid staining and quantitation

Lipid staining was performed using Oil Red O dyeing solution as described previously [37]. Worms were washed off the NGM or RNAi plates and incubated in PBS (phosphate-buffered saline) buffer for 30 min on a shaker at room temperature. The worms were then fixed in Modified Ruvkun’s Witches Brew (MRWB) buffer containing 1% paraformaldehyde. After three rounds of freezing/thawing, the worms were dehydrated in 60% isopropanol followed by addition of saturated Oil Red O (Sigma, USA) solution. Fixed worms were incubated overnight on a shaker at room temperature, mounted on slides and viewed using a microscope with differential interference contrast optics (Nikon, Japan).

For quantification of Oil Red O staining, using ImageJ image processing software, we separated out each color image into its Red, Green and Blue (RGB) channel components. As it has been reported that Oil Red O absorbs light at 510 nm, we used the green channel for further analysis [38]. We measured the average pixel intensity for a 40-pixel radius immediately behind the pharynx of each animal. In addition, we measured a 40-pixel radius of the background, which was later subtracted from the values obtained from the staining. A minimum of nine animals was measured for each strain, and we repeated the experiments two additional times. Significance was determined by Student’s *t*-test.

## Results

### Structural characterization of *Acan*-Gal-1

**Fig. 1:**
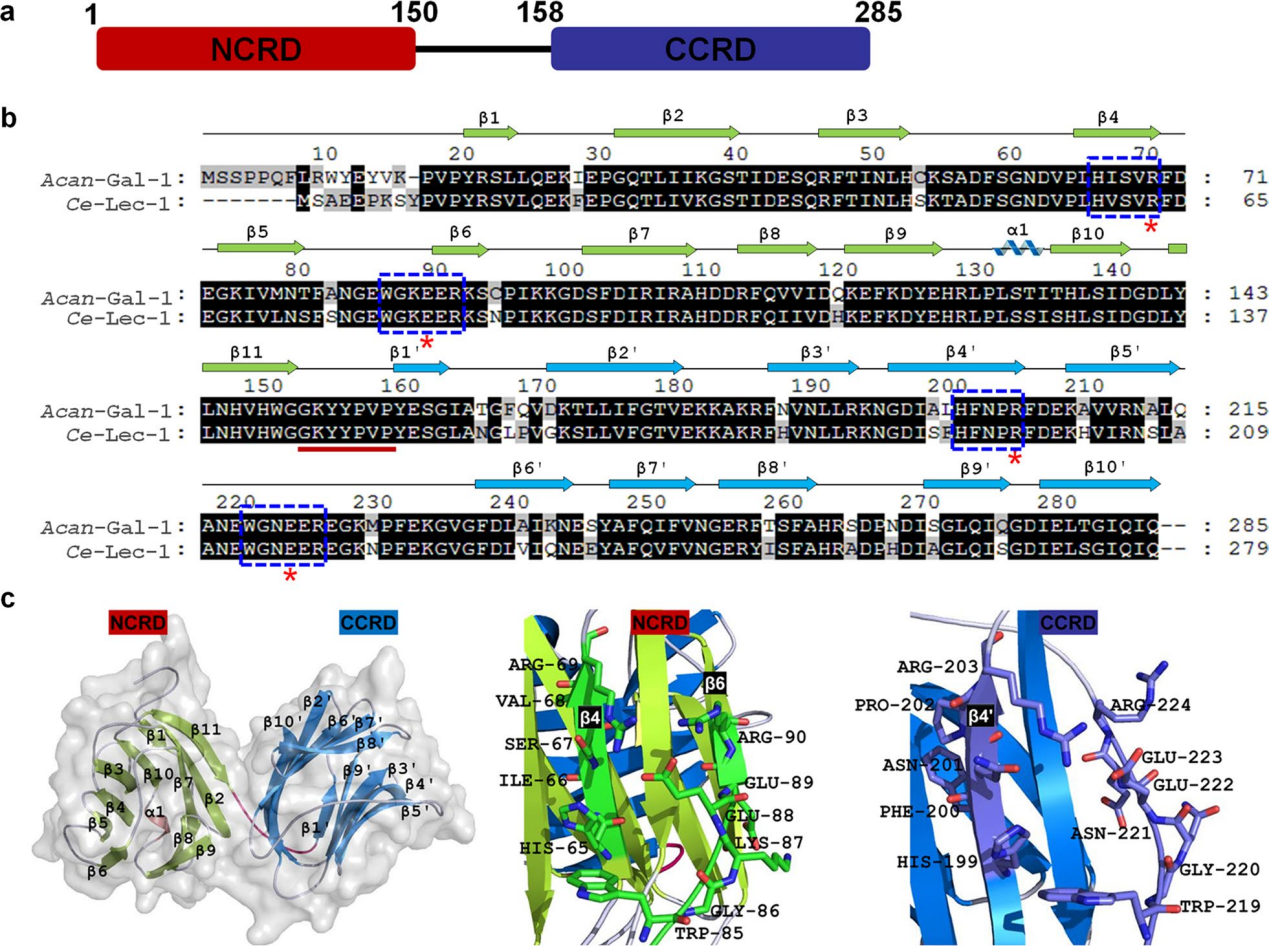
Structures and sequence analysis of *Acan-*Gal-1. **a** Schematic diagram showing the domains of *Acan-*Gal-1 (residues 1–285). **b** Alignment of amino acid sequences of *Acan-*Gal-1 from *A. cantonensis* with that from *C. elegans* and the secondary structures. The accession number of *Ce*-Lec-1 sequence available from the current database is: NP_001370038.1. Identical and similar residues are shown in black and gray blocks, respectively. Secondary elements are shown by coil (α-helix) and arrows (green indicates β-sheets of NCRD and blue indicates those of CCRD). The short linker between NCRD and CCRD is indicated by a red line. The conserved motifs H*XXX*R and WG*X*EER are indicated by blue boxes. The critical amino acids for recognizing carbohydrate binding are indicated by red stars. **c** Predicted tertiary structure of *Acan-*Gal-1, H*XXX*R motif and WG*X*EER motif in NCRD and CCRD, respectively

To characterize the structure of *Acan-*Gal-1, amino acid sequence alignment and structural analysis were performed. Full-length *Acan-*Gal-1 was composed of 285 amino acids, containing N-terminal carbohydrate recognition domain (CRD) (NCRD, residues 1–150) and C-terminal CRD (CCRD, residues 158–285) in the manner of tandem repeat, and a short linker (residues 151–157) held them together (Fig. 1a). The amino acid sequence was aligned with *Ce*-Lec-1 (Fig. 1b). The results showed that *Acan-*Gal-1 had a similarity of 84.4% to *Ce*-Lec-1. The conserved motifs H*XXX*R and WG*X*EER, involved with carbohydrate binding sites [35], were located in both NCRD and CCRD. The charged Arg^69^/Arg^203^ and Glu^88^/Glu^222^ were conserved in the motifs, which are critical amino acids for recognizing carbohydrate binding and affect protein folding and structure [35]. The linker “GKYYPVP,” shorter than other tandem repeat-type galectins such as galectin-9, which are flexible and susceptible to proteolysis [20, 39, 40], may indicate the structural stability of *Acan-*Gal-1. Structural analysis from homology models revealed that the NCRD of *Acan-*Gal-1 possessed 11 β-sheets and a small α-helix located between β9 and β10, whereas the CCRD contained 10 β-sheets and no α-helix (Fig. 1b, c). The conserved motifs H*XXX*R and WG*X*EER, the carbohydrate binding sites, were located in the concave surface surrounded by β4 and β6 of NCRD, and those were also located in the surface formed by β4′ and the loop between β5′ and β6′ of CCRD (Fig. 1c).

### Expression patterns of *Acan*-Gal-1

**Fig. 2:**
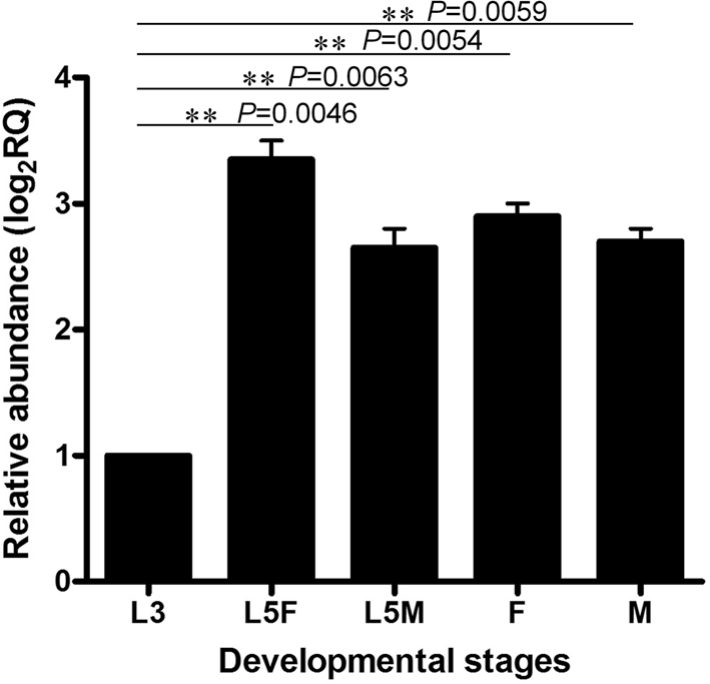
Transcriptional profile of *Acan-gal-1* in different developmental stages (L3, L5 and adult) and genders [females (F) and males (M)] of *A. cantonensis*, determined by real-time PCR analysis. Data shown are mean ± SEM derived from three technical replicates with two biological replicates. Relative transcription of the *Acan-gal-1* gene in each sample was calculated by normalization of the raw data, followed by the determination of abundance relative to the 18S ribosomal RNA gene (GenBank: AY295804), which served as an internal loading control. Statistical analysis was conducted using a one-way ANOVA. *P < 0.05; **P < 0.01

To determine the relative abundance of *Acan-gal-1* transcript in different developmental stages (L3, L5 and adult) and genders [females (F) and males (M)] of the life cycle of *A. cantonensis*, qRT-PCR was performed with the 18S ribosomal RNA gene as an internal loading control. The results showed that *Acan-gal-1* was transcribed in larval and adult developmental stages examined in different levels (Fig. 2; Additional file 2: Table S2). The expressions of *Acan-gal-1* were greatly upregulated in both L5 and adult compared with those in L3, whereas the expression levels were not significantly changed among L5, adult and different genders. This might indicate the important roles of *Acan*-Gal-1 in L5 and adults, which reside in mammals, such as humans and rats, where a full immune system exists.

**Fig. 3:**
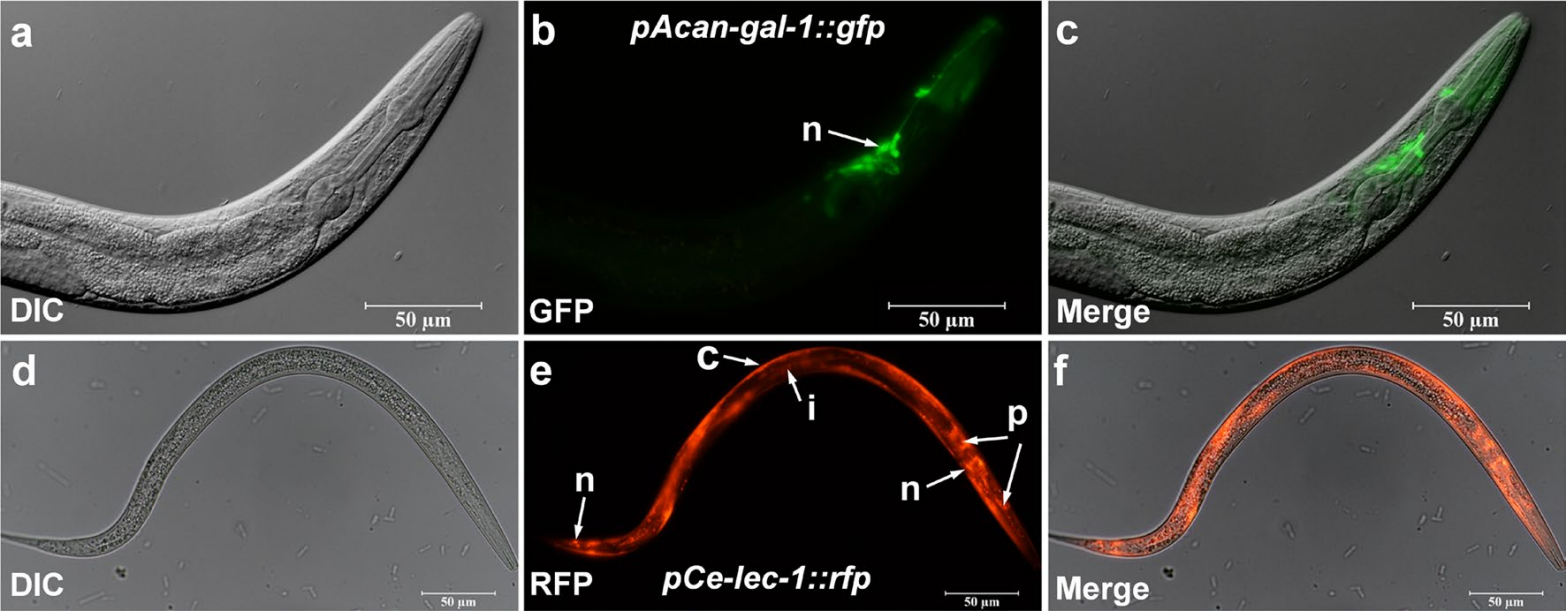
Expression pattern of *A. cantonensis Acan-gal-1* promoter in *C. elegans*. **a**–**c** Promoter activity of *Acan-gal-1* in *C. elegans*. *pAcan-gal-1::gfp* is only expressed in pharyngeal nerves of *C. elegans*. **d**–**f** Promoter activity of *Ce-lec-1* in *C. elegans*. *pCe-lec-1::rfp* is expressed widely. Arrows indicate the following tissues: c, cuticle; i, intestine; n, neuron; p, pharynx

Because of the lack of functional genetic and in vitro culture methods, it is not possible to detect the functions of *Acan*-Gal-1 directly in *A. cantonensis*. Here, *C. elegans*, proposed by numerous authors as a general model for many aspects of basic molecular, cellular and developmental biology in the less tractable parasitic nematodes [41–43], was used to investigate the anatomical expression patterns of *Acan*-Gal-1. Wild-type *C. elegans* (N2 strain) were transformed with the construct *pAcan-gal-1::gfp* and *pCe-lec-1::rfp*, respectively. The results showed that GFP under the promoter *pAcan-gal-1* was only expressed in pharyngeal neurons of *C. elegans* (Fig. 3a–c), in contrast to the situation in worms expressing *pCe-lec-1::rfp*, where RFP was mainly localized in cuticle and less in intestine, nervous system and pharynx (Fig. 3d–f). This result was in agreement with the previous report [32].

### Cross-species expressions of *Acan*-Gal-1 in *C. elegans* worms

The different activities of *pAcan-gal-1* and *pCe-lec-1* might be due to the heterologous expression, with low promoter sequence similarity (data not shown). Therefore, *pCe-lec-1* was used as the promoter in this research on the functions of *Acan*-Gal-1 in *C. elegans*.

**Fig. 4:**
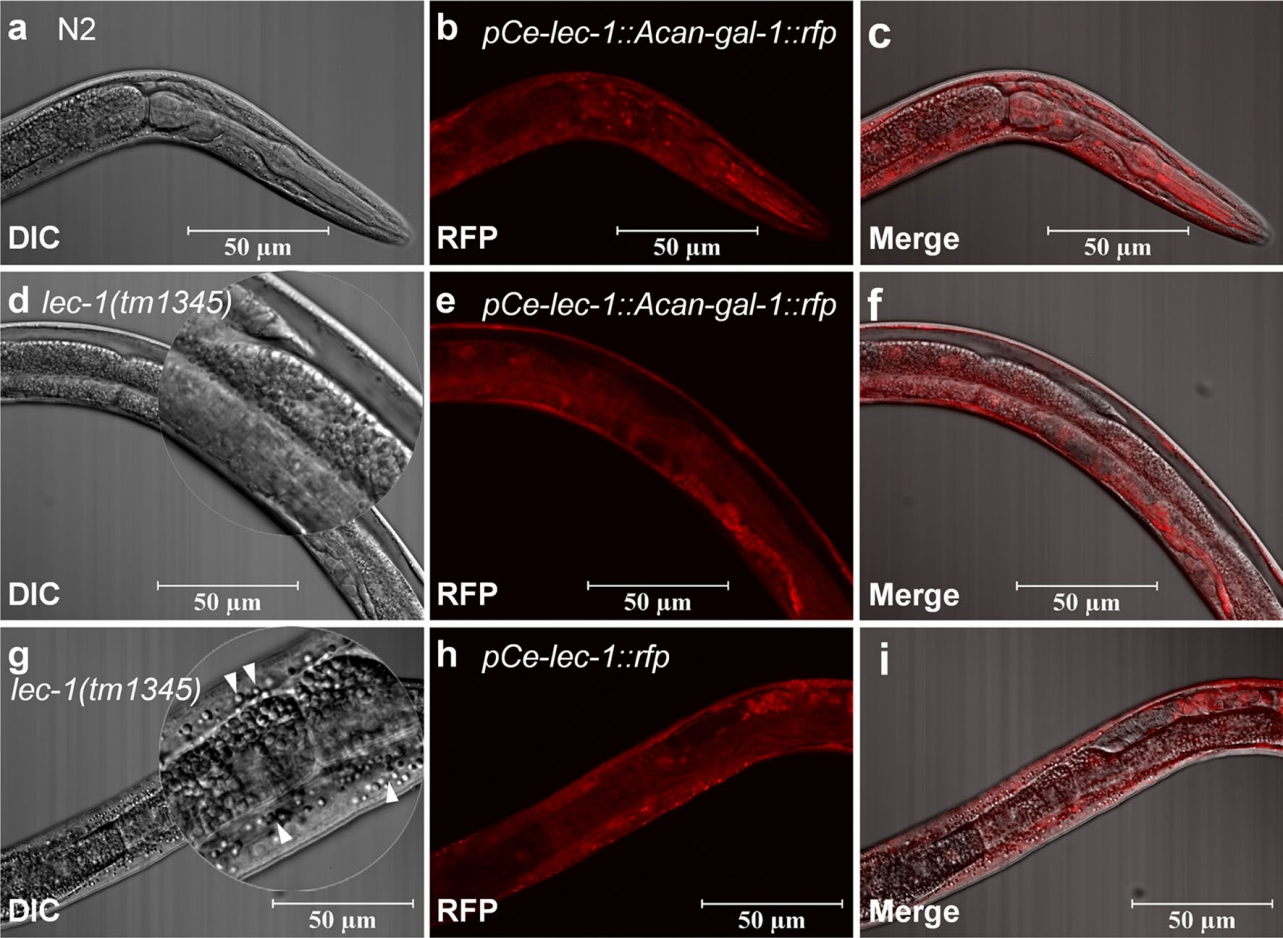
Cross-species expressions of *Acan*-Gal-1 in N2 and the *lec-1* deletion mutant worms. **a**–**c** Expression of *pCe-lec-1::Acan-gal-1::rfp* in N2 worm. **d**–**f** Expression of *pCe-lec-1::Acan-gal-1::rfp* in *lec-1* deletion mutant worm. **g**–**i** Expression of *pCe-lec-1::rfp* in *lec-1* deletion mutant worm. White arrowheads indicate the lipid drops

To clarify the role of *Acan*-Gal-1, cross-species expression in *C. elegans* was performed. The expressing constructs containing *Acan-gal-1::rfp* coding sequences driven by *Ce-lec-1* promoter were used to transform *C. elegans* strains N2, *lec-1* (*tm1345*), *ced-3* (*ok2734*) and *fat-6;fat-7* (BX156), respectively, and *pCe-lec-1::rfp* transforming was used as control. In worms transformed with *pCe-lec-1::Acan-gal-1::rfp*, RFP was expressed widely, and mainly in cuticle (Fig. 4), consistent with the *pCe-lec-1::rfp* expression pattern.

Morphological changes were further detected under a light microscope, and we found the level of lipid storage in *lec-1* mutant worms expressing *pCe-lec-1::Acan-gal-1::rfp* was lower (Fig. 4d) than that in *lec-1* mutant worms expressing *pCe-lec-1::rfp* (Fig. 4g). This might suggest the function of *Acan*-Gal-1 in reducing lipid deposition in *C. elegans*.

### Functional role of *Acan*-Gal-1 in lipid storage

**Fig. 5:**
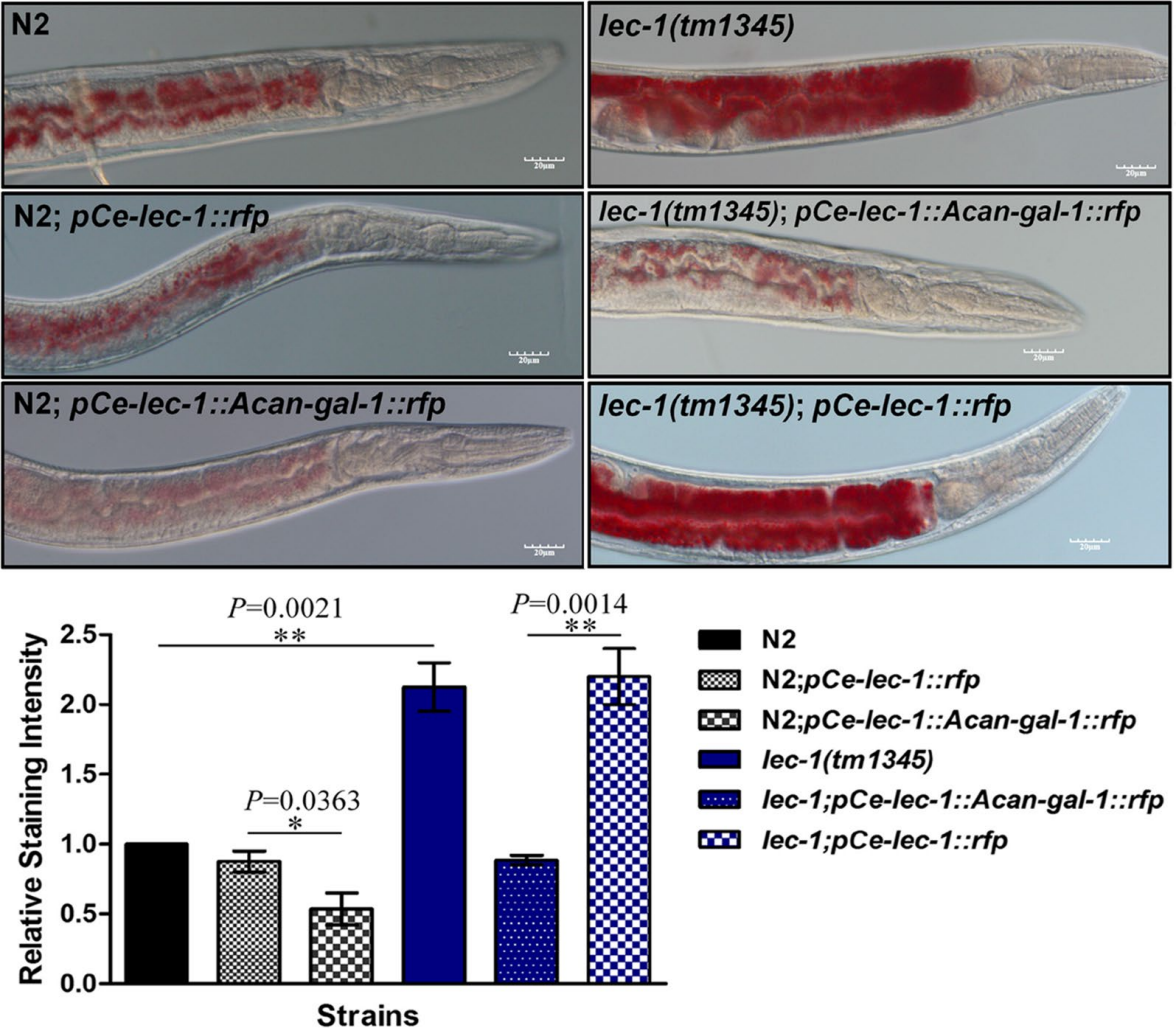
Upregulated *Acan*-Gal-1 functioned in regulating lipid deposition. Oil Red O fat staining was performed in *C. elegans* strains: N2, N2 expressing *pCe-lec-1::rfp*, N2 expressing *pCe-lec-1::Acan-gal-1::rfp*, *lec-1(tm1345)* mutant, *lec-1(tm1345)* mutant expressing *pCe-lec-1::rfp* and *lec-1(tm1345)* mutant expressing *pCe-lec-1::Acan-gal-1::rfp*. Quantification of Oil Red O staining was performed using ImageJ image processing software. The error bars indicate standard deviation. *P < 0.05; **P < 0.01

To explore the function of *Acan*-Gal-1 in reducing lipid deposition, Oil Red O fat staining was performed in *C. elegans*. The results showed that *lec-1* mutant worms stored significantly more lipid than N2 worms, and this lipid accumulation phenotype could be rescued by expressing *pCe-lec-1::Acan-gal-1::rfp* in *lec-1* mutant worms. The N2 worms expressing *pCe-lec-1::Acan-gal-1::rfp* exhibited much less lipid storage than the N2 worms expressing *pCe-lec-1::rfp* (Fig. 5; Additional file 2: Table S2). This might suggest the function of *Acan*-Gal-1 in reducing lipid deposition in *C. elegans*.

### Functional role of *Acan-Gal-1* in resisting oxidative stress

**Fig. 6:**
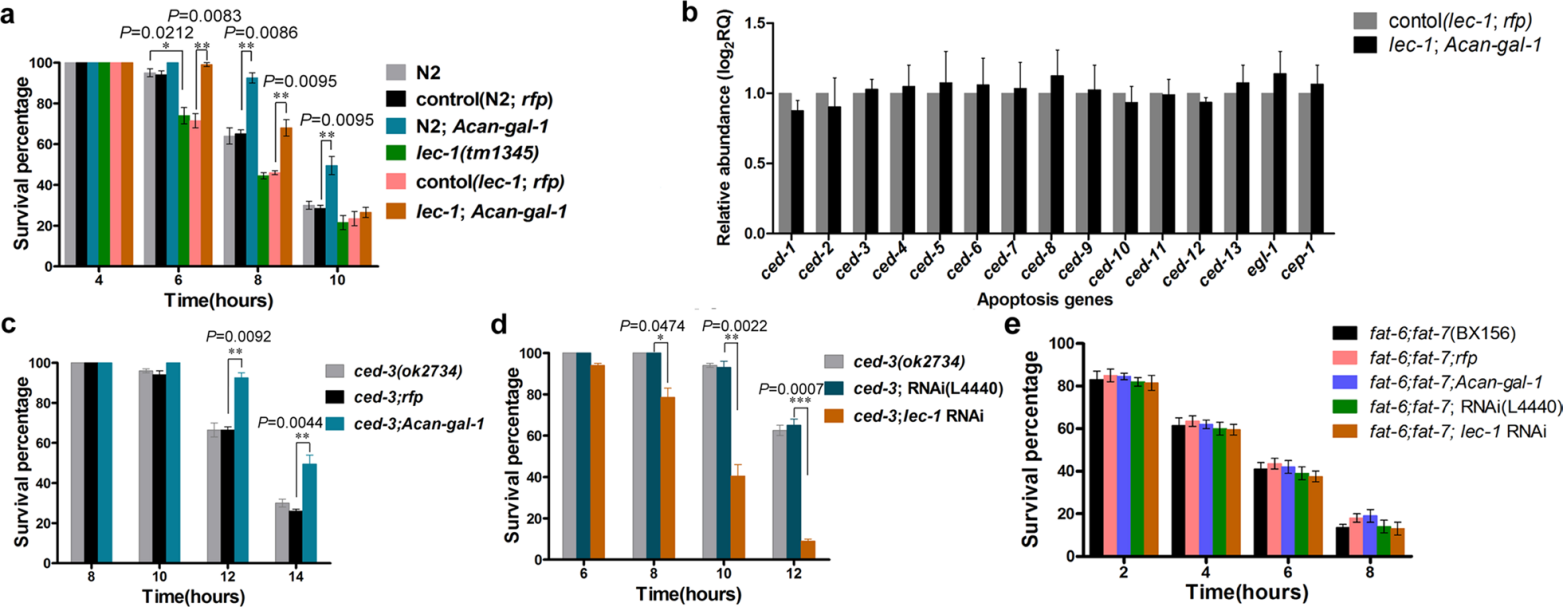
Upregulated *Acan*-Gal-1 plays a defensive role against oxidative stress. **a** Oxidative stress assays using H_2_O_2_ in N2 and *lec-1* mutant worms expressing *pCe-lec-1::Acan-gal-1::rfp*. **b** Relative expression levels of apoptosis genes in *lec-1* mutant worms expressing *pCe-lec-1::rfp* and in *lec-1* mutant worms expressing *pCe-lec-1::Acan-gal-1::rfp*. **c** Oxidative stress assays using H_2_O_2_ in *ced-3* mutant worms expressing *pCe-lec-1::Acan-gal-1::rfp* or *pCe-lec-1::rfp*. **d** Oxidative stress assays using H_2_O_2_ in *ced-3* mutant worms with *lec-1* RNAi. **e** Oxidative stress assays using H_2_O_2_ in *fat-6;fat-7* double-mutant worms expressing *pCe-lec-1::Acan-gal-1::rfp* or with *lec-1* RNAi. The worms were counted as described in “Methods” section. The error bars indicate standard deviation. *P < 0.05; **P < 0.01; ***P < 0.001

LEC-1 plays a defensive role against damage due to oxidative stress in *C. elegans*, and *lec-1* (*tm1345*) worms show susceptibility to H_2_O_2_ [33]. To investigate the role of *Acan*-Gal-1 in regulating oxidative stress resistance, we performed oxidative stress assays using H_2_O_2_. We found that the incidence of rapid death among *lec-1* deletion mutants was significantly higher than that among the N2 worms, and this oxidative stress susceptibility phenotype could be rescued by expressing *pCe-lec-1::Acan-gal-1::rfp* in *lec-1* mutant worms. The N2 worms expressing *pCe-lec-1::Acan-gal-1::rfp* were significantly more resistant to H_2_O_2_ than the N2 worms expressing *pCe-lec-1::rfp* (Fig. 6a; Additional file 2: Table S2). This might suggest the regulating role of *Acan*-Gal-1 in increasing oxidative stress tolerance.

As we have demonstrated that downregulated *Acan*-RPS-30 in *A. cantonensis* L5 can resist oxidative stress damage by inhibiting worm apoptosis [17], and oxidative stress is thought to be one of the major factors that promote apoptosis [44], we next determined whether *Acan*-Gal-1 increased oxidative stress tolerance via inhibiting worm apoptosis. Then, the expression levels of apoptosis genes were detected in *C. elegans*. The results showed that all the apoptosis genes were not significantly changed in *lec-1* mutant worms expressing *pCe-lec-1::Acan-gal-1::rfp* compared with those in *lec-1* mutant worms expressing *pCe-lec-1::rfp* (Fig. 6b). This indicated that expressing *Acan*-Gal-1 could not regulate apoptosis genes in transcriptional level.

*ced-3* is the final executive gene in the core apoptosis pathway in *C. elegans*, and apoptosis is inhibited in *ced-3* mutant worms [45]. Therefore, oxidative stress assays were further performed in *ced-3* mutant worms to detect the effects of expressing *Acan*-Gal-1 on the oxidative stress damage. The results showed that the incidence of rapid death among *ced-3* mutant worms expressing *pCe-lec-1::Acan-gal-1::rfp* was significantly lower than that among *ced-3* mutant worms expressing *pCe-lec-1::rfp* (Fig. 6c; Additional file 2: Table S2), whereas *lec-1* RNAi made *ced-3* mutant worms greatly more susceptibility to oxidative stress (Fig. 6d; Additional file 2: Table S2). This indicated that *Acan*-Gal-1 regulated the oxidative stress resistance was not via apoptosis in *C. elegans*.

Fatty acid metabolism is involved in the oxidative stress resistance in *C. elegans*, and reduction of fat storage makes worms strongly resistant to oxidative stress [46, 47]. We next investigated whether *Acan*-Gal-1 increased oxidative stress tolerance via reducing lipid deposition in *C. elegans*. *fat-6;fat-7* double-mutant worm was selected because *fat-6* and *fat-7* genes encode stearoyl-CoA desaturases, which are key lipogenic enzymes, and *fat-6;fat-7* double-mutant worms have decreased fat storage [48]. Then, oxidative stress assays were performed to determine the effects of expressing *Acan*-Gal-1 on the oxidative stress damage in *fat-6*; *fat-7* double-mutant worms. The results showed that the incidence of rapid death was not significantly changed among *fat-6;fat-7* double-mutant worms expressing *pCe-lec-1::Acan-gal-1::rfp* compared with that among *fat-6;fat-7* double-mutant worms expressing *pCe-lec-1::rfp* (Fig. 6e). The susceptibility to oxidative stress was not influenced by *lec-1* RNAi in *fat-6;fat-7* double-mutant worms (Fig. 6e). Expression of either *Acan*-Gal-1 or *lec-1* RNAi had no significant effect on oxidative stress resistance in *fat-6;fat-7* double-mutant worms, where fat storage is reduced dramatically. This might indicate the function of *Acan*-Gal-1 in increasing oxidative stress tolerance was related to fat storage reduction in *C. elegans*.

## Discussion

The parasitic nematode *A. cantonensis* L5, residing in human cerebrospinal fluid, can cause eosinophilic meningitis, primarily due to eosinophil-inducing tissue inflammatory responses. Eosinophils, as well-equipped immune cells, can be recruited from the circulation into inflammatory sites in response to helminthic infections to play a role in protecting the host against the infection; at the same time, they cause some tissue damage when the inflammatory response is serious. Eosinophil peroxidase, as a cytotoxic granular protein in eosinophils, plays a crucial role in killing helminths [49]. A high level of the enzyme complex that generates superoxide is expressed in eosinophils, and superoxide anions are produced in response to helminth-derived immunomodulating agents. Therefore, eosinophils are robust producers of extracellular superoxide. In contrast, helminthic worms have evolved to attenuate the oxidative stress damage from eosinophils for their survival in hosts, such as inducing apoptosis of eosinophils [50, 51] and increasing oxidative stress resistance of the worm itself [17]. We have demonstrated that *Acan*-Gal-1, upregulated in *A. cantonensis* L5, which resides in humans with more sophisticated immune systems, could induce apoptosis of macrophages extracellularly when secreted by cells via an unconventional mechanism [24]. *Acan*-Gal-1 could also function intracellularly [25, 26, 33]. In this study, we investigated the intracellular functions of *Acan*-Gal-1in protecting worms from immune attacks in hosts.

Lack of effective genetic manipulation in parasitic nematodes and *A. cantonensis* L5 in in vitro culture methods makes it impossible to study the in vivo functions of *Acan*-Gal-1 in *A. cantonensis*. The free-living nematode *C. elegans*, which belongs to clade V in the phylogenetic relationship, like *A. cantonensis* [17, 41], has been used widely in scientific research as the model organism, especially for exploring gene functions of parasitic nematodes, such as *Haemonchus contortus*, *Strongyloides stercoralis*, etc. [52–54]. Therefore, in this study, it was employed as a surrogate to explore the intracellular functions through cross-species expression of *Acan*-Gal-1.

Here, the expression pattern of *Acan-gal-1* promoter was not consistent with that of *Ce-lec-1* promoter in *C. elegans*. This might be because of the low promoter sequence similarity between *Acan-gal-1* promoter and *Ce-lec-1* promoter. There are many examples of heterologous expression in *C. elegans*, such as *daf-2*, *daf-16* and *rps-30* from *Haemonchus contortus* [37, 52, 54], *daf-16* from *Strongyloides stercoralis*, et al*.* [53] and GFP or RFP mainly expressed in the intestinal tract or nervous system, where heterologous expression easily occurs. For example, in the parasitic nematode *Haemonchus contortus*, *pHc-rps30::gfp* is expressed in the distal intestine of *C. elegans* using cross-species expression, in contrast to *pCe-rps30::gfp*, expressed ubiquitously. *Hc-rps30* is also expressed ubiquitously in *H. contortus*, consistent with the expression pattern of *pCe-rps30::gfp* in *C. elegans*, when it is localized using whole-mount in situ hybridization [37]. The expression pattern of *Hc-rps30* in *Haemonchus contortus* is consistent with that of *Ce-rps30* in *C. elegans*, though cross-species expression of *Hc-rps30* is very different. In this study, we tried to determine the expression pattern of *Acan-gal-1* in *Angiostrongylus catonensis* using whole-mount in situ hybridization. Unfortunately, *A. cantonensis* L5 worms were too big, with thick cuticle and dark color, and the location was not successful. However, in our opinion, the expression pattern of *Acan-gal-1* in *Angiostrongylus catonensis* should be consistent with that of *Ce-rps30* in *C. elegans*, and it was reasonable to express *Acan-gal-1* under *Ce-lec-1* promoter in *C. elegans*, referring to previous studies and the characterization of heterologous expression in *C. elegans*.

Exploring the intracellular functions through cross-species expression showed that *Acan*-Gal-1 could increase the oxidative stress tolerance in *C. elegans*. This might indicate the regulating function of *Acan*-Gal-1 in attenuating eosinophil-mediated immune attack upon *A. cantonensis* L5 worms in the central nervous system of human by its upregulated expression. However, L3 worms, with low levels of *Acan*-*gal-1*, reside in intermediate hosts (e.g. *P. canaliculata*) in which the immune system is less sophisticated than that in mammalians, and the immune attack may be weaker; there may even be no eosinophil-mediated superoxide attack in snails. Furthermore, the expression levels were significantly upregulated in both L5 and adults, both of which reside in mammalians such as humans and rats, respectively, who have more sophisticated immune systems. Upregulated *Acan*-Gal-1 might protect L5 and adults from attacks of inflammatory response by both promoting immune cells apoptosis and increasing oxidative stress tolerance of the worm itself.

In this study, we found *Acan*-Gal-1 could function in both reducing fat storage and increasing oxidative stress tolerance in *C. elegans*. Fatty acid metabolism is involved in stress-resistance mechanisms, and reduction of fat storage makes worms resistant to oxidative stress [46, 47]. Unsaturated fatty acids are readily oxidized by intercellular reactive oxygen species [55] and can act as intracellular scavengers [46]. Therefore, the function of *Acan*-Gal-1 in reducing fat storage might be involved in increasing oxidative stress tolerance. To explore this, fat storage reduced *C. elegans* worms, *fat-6;fat-7* double mutants, were used to test the function of *Acan*-Gal-1 in oxidative stress resistance in worms, where fat storage is reduced dramatically. Interestingly, expressing neither *Acan*-Gal-1 nor *lec-1* RNAi significantly influenced the oxidative stress resistance in *fat-6;fat-7* double-mutant worms. This might further suggest *Acan*-Gal-1 functioned in increasing oxidative stress tolerance via reducing lipid deposition in *C. elegans*.

## Conclusions

Here, we determined the structural characterization and functions of *Acan*-Gal-1 from *A. cantonensis*. *Acan*-Gal-1 could promote worms' resistance to oxidative stress by reducing fat deposition. Our findings may further reveal the mechanism of *A. cantonensis* L5 worms surviving inflammatory responses of the human central nervous system.

## Supplementary Information

**Additional file 1: Table S1.** List of primers used in this study.

**Additional file 2: Table S2.** Statistical comparisons presented in figures.

## Data Availability

Data supporting the conclusions of this article are included within the article and its additional files. The datasets used in the present study are available from the corresponding author upon reasonable request.

## Abbreviations

ZJ: Zhejiang
Gly: Glycine
p.i: Post-infection
PBS: Phosphate-buffered saline
PDB: Protein data bank
GFP: Green fluorescent protein
RFP: Red fluorescent protein
bp: Base pair
UTR: Untranslated region
CEP-1: *C. elegans* P-53-like protein
CED: Cell death abnormality
EGL: EGg laying defective
ZJ: Zhejiang
RT-PCR: Real-time PCR
RGB: Red, green and blue
RNAi: RNA interference
CoA: Coenzyme A
