# Supplementary material for: Upregulated galectin-1 in Angiostrongylus cantonensis L5 reduces body fat and increases oxidative stress tolerance
Source: Parasit Vectors. 2022 Feb 5;15:46. doi: 10.1186/s13071-022-05171-4 (PMC8817484; doi:10.1186/s13071-022-05171-4)
Supplement: Supplementary file 1 — Additional file 1: Table S1. List of primers used in this study. [file 13071_2022_5171_MOESM1_ESM.doc]

**Additional file 1: Table S1. List of primers used in this study.**

| Primer name | Gene amplified | Used for | | | Sequence (5`-3`) |
| --- | --- | --- | --- | --- | --- |
| acgal-1F | Acan-gal-1 | PCR | | | ATGTCGTCTCCTCCACAGTTT |
| acgal-1R | Acan-gal-1 | PCR | | | CTACTGAATTTGAATGCCGG |
| p acgal-1F | *p* Acan-gal-1 | expression patterns | | | cgGGATCCTGACGTGGTTGAAGTGTGA |
| p acgal-1R | *p* Acan-gal-1 | expression patterns | | | tccCCCGGGAGTACGATCGTTCAACAGCT |
| p Ce-lec-1F | *p* Ce-lec-1 | expression patterns | | | cgGGATCCCCTTTGTGCACTGGCGAATTC |
| p Ce-lec-1-R | *p* Ce-lec-1 | expression patterns | | | tccCCCGGGACTAGATGGACGAGGTGAA |
| lec-1F | Ce-lec-1 | RNAi | | | tgCTCTAGAATGAGCACCATGAATGCAGC |
| lec-1R | Ce-lec-1 | RNAi | | | ggGGTACCGAGATTGTCAAAATTGAGGC |
| real-egl-1-F | egl-1 | RT-PCR | | | AGCACCATGAATGCAGCCAA |
| real-egl-1-R | egl-1 | RT-PCR | | | GCTCATGTATCAACGGACTC |
| real-cep-1-F | cep-1 | RT-PCR | | | GCGTCGGTCTCGTCGTCTTT |
| real-cep-1-R | cep-1 | RT-PCR | | | TCGCTCGGCTTCTTACGACA |
| real- Ac-18S-F | *18srna* | RT-PCR | | | TGGATCTGAGTTGCATGCA |
| real- Ac-18S-R | *18srna* | RT-PCR | | | CGCGCAGGGATACGAATGC |
| real-ced-1-F | *ced-1* | | RT-PCR | TGAGCCGGGAAAATGTGAATG | |
| real-ced-1-R | *ced-1* | | RT-PCR | CATCGTTCTCCTTGAAATCCAC | |
| real-ced-2-F | *ced-2* | | RT-PCR | AATCGCGAATCAGTCGTTTCC | |
| real-ced-2-R | *ced-2* | | RT-PCR | CACCAATCCTGGTTCGTTTTTG | |
| real-ced-3-F | *ced-3* | | RT-PCR | CGACGAGAAAACCATGTACAGA | |
| real-ced-3-R | *ced-3* | | RT-PCR | GGCAAAGTCTCGAATTGTCAG | |
| real-ced-4-F | *ced-4* | | RT-PCR | CGCAATGGCTCTTCAAAGATG | |
| real-ced-4-R | *ced-4* | | RT-PCR | GCATTCGTTTTCCACTGAGAAG | |
| real-ced-5-F | *ced-5* | | RT-PCR | CGTGGAATGTGTGGATAGTCTT | |
| real-ced-5-R | *ced-5* | | RT-PCR | GCATTGTGGCATGTCGAAGTA | |
| real-ced-6-F | *ced-6* | | RT-PCR | GGATTCATCCGCCAGATTATC | |
| real-ced-6-R | *ced-6* | | RT-PCR | AGTGGAAATTCGCATCTCGATC | |
| real-ced-7-F | *ced-7* | | RT-PCR | GTGAAGAACAACTGCTGACTG | |
| real-ced-7-R | *ced-7* | | RT-PCR | CTTGGTCCATACGGATTGTAAC | |
| real-ced-8-F | *ced-8* | | RT-PCR | GCTGAAAGGGATGCAACATTG | |
| real-ced-8-R | *ced-8* | | RT-PCR | GTAACGAGCGGTTTTGAACAAC | |
| real-ced-9-F | *ced-9* | | RT-PCR | CACGCGGAAAATTTTGAGACC | |
| real-ced-9-R | *ced-9* | | RT-PCR | CCACGGATTCCATCATTTTTGC | |
| real-ced-10-F | *ced-10* | | RT-PCR | ATGCAAGCGATCAAATGTGTCG | |
| real-ced-10-R | *ced-10* | | RT-PCR | CGATCGTAATCTTCCTGTCCA | |
| real-ced-11-F | *ced-11* | | RT-PCR | TTCCATCCGAACCAATGAGTC | |
| real-ced-11-R | *ced-11* | | RT-PCR | TGGCACATAATTCACAGCTGTC | |
| real-ced-12-F | *ced-12* | | RT-PCR | TGTGCATCACCGGATCATTATG | |
| real-ced-12-R | *ced-12* | | RT-PCR | TACAGCTAATTCCAGCATACTTG | |
| real-ced-13-F | *ced-13* | | RT-PCR | TGATGTCGTACAAGCGTGATG | |
| real-ced-13-R | *ced-13* | | RT-PCR | TCAAACTCGTCGCACATAACTG | |

RT-PCR, real-time PCR; RNAi, RNA interference.
