# Supplementary material for: Upregulated galectin-1 in Angiostrongylus cantonensis L5 reduces body fat and increases oxidative stress tolerance
Source: Parasit Vectors. 2022 Feb 5;15:46. doi: 10.1186/s13071-022-05171-4 (PMC8817484; doi:10.1186/s13071-022-05171-4)
Supplement: Supplementary file 2 — Additional file 2: Table S2. Statistical comparisons presented in figures. [file 13071_2022_5171_MOESM2_ESM.docx]

**Additional file 2: Table S2.** Statistical comparisons presented in the figures

**Figure 2**

**ANOVA: F(4,10)=55.326, *P*<0.001**

| Comparing groups | *P* value corrected by LSD |
| --- | --- |
| L3 vs L5F | 0.0046 |
| L3 vs L5M | 0.0063 |
| L3 vs F | 0.0054 |
| L3 vs M | 0.0059 |

**Figure 5b**

| Comparing groups | *t*-test statistic and *P*-value |
| --- | --- |
| N2; *rfp* vs N2; *Acan-gal-1* | t(4) = 3.223, *P*=0.0363 |
| N2 vs *lec-1(tm1345)* | t(4) = -26.347, *P*=0.0021 |
| *lec-1;* *Acan-gal-1* vs *lec-1; rfp* | t(4) = -27. 937, *P*=0.0014 |

**Figure 6a**

| Time (h) | Comparing groups | *t*-test statistic and *P*-value |
| --- | --- | --- |
| 6 | N2 vs *lec-1(tm1345)* | t(4) =4.114, *P*=0.0212 |
|  | *lec-1*;*rfp* vs *lec-1*;*Acan-gal-1* | t(4) =-5.334, *P*=0.0083 |
| 8 | N2;*rfp* vs N2;*Acan-gal-1* | t(4) =-4.803, *P*=0.0086 |
|  | *lec-1*;*rfp* vs *lec-1*;*Acan-gal-1* | t(4) =-4.886, *P*=0.0095 |
| 10 | N2;*rfp* vs N2;*Acan-gal-1* | t(4) =-7.543, *P*=0.0053 |

**Figure 6c**

| Time (h) | Comparing groups | *t*-test statistic and *P*-value |
| --- | --- | --- |
| 12 | *ced-3;rfp* vs *ced-3; Acan-gal-1* | t(4) =-4.602, *P*=0.0092 |
| 14 | *ced-3;rfp* vs *ced-3; Acan-gal-1* | t(4) =-8.021, *P*=0.0044 |

**Figure 6d**

| Time (h) | Comparing groups | *t*-test statistic and *P*-value |
| --- | --- | --- |
| 8 | *ced-3;*RNAi(L4440) vs *ced-3;lec-1* RNAi | t(4) =3.82, *P*=0.0474 |
| 10 | *ced-3;*RNAi(L4440) vs *ced-3;lec-1* RNAi | t(4) =-10.576, *P*=0.0022 |
| 12 | *ced-3;*RNAi(L4440) vs *ced-3;lec-1* RNAi | t(4) =-28.387, *P*=0.0007 |
